# The caregiver’s experience of childhood cancer treatment in South Africa

**DOI:** 10.1080/20523211.2024.2312382

**Published:** 2024-02-29

**Authors:** I. R. Joosse, H. A. van den Ham, A. K. Mantel-Teeuwisse, F. Suleman

**Affiliations:** a Utrecht WHO Collaborating Centre for Pharmaceutical Policy and Regulation, Division of Pharmacoepidemiology and Clinical Pharmacology, Utrecht Institute for Pharmaceutical Sciences (UIPS), Utrecht University, Utrecht, The Netherlands; b WHO Collaborating Centre for Pharmaceutical Policy and Evidence Based Practice, School of Health Sciences, University of KwaZulu-Natal, Durban, South Africa

**Keywords:** Childhood cancer, cancer treatment, caregiver experiences, focus group, financial strain, South Africa

## Abstract

**Background:**

This study explored the treatment-related, financial and psychological experiences of caregivers during cancer treatment of their children in South Africa’s (SA) public and private sectors.

**Methods:**

In this exploratory study, three focus groups were conducted with caregivers of children undergoing cancer treatment in SA’s public healthcare sector. A fourth small focus group with two parents in the private sector was conducted online. A mixed-methods approach was employed using a combination of thematic analysis and grounded theory.

**Results:**

Of the 20 public sector caregivers, many expressed frustration at the number of visits to primary healthcare clinics before being referred. Caregivers had difficulties coping with and accepting the diagnosis, alongside managing continued care for the child and other children at home. Support received by family and community members was varied. Financial strain was an important concern. The two private sector parents indicated greater levels of support and no financial hardship, but expressed similar levels of emotional stress.

**Conclusion:**

These caregiver experiences indicate that improvements are urgently needed in the recognition of childhood cancer symptoms at primary healthcare level in SA. They also highlight a need for increased financial support from government through social grants, travel allowances and nutritional support.

## Background

With current survival rates of approximately 50% (Ward et al., 2019; Stones et al., 2014), South Africa is committed to achieving the World Health Organization’s (WHO) Global Initiative for Childhood Cancer (GICC) target of at least 60% overall survival for children (WHO, 2021) and improving the lives of those living with or surviving cancer (National Department of Health, 2017). To achieve this, the need for health systems strengthening and addressing the persistent health disparities (Harris et al., 2011; Gordon et al., 2020) were recognised as priorities within the National Cancer Strategic Framework (NCSF) (National Department of Health, 2017).

To effectively inform policy development, an understanding of the barriers and facilitators in access to childhood cancer treatment is required. Therefore, a comprehensive health system analysis, with a particular focus on medicines, was undertaken (Joosse et al., 2023; Joosse et al., n.d). Key barriers identified – through a study of policy documents and interviews with stakeholders in both the public and the private healthcare setting – included a lack of political priority given to childhood cancer (medicines), novel therapeutics not seeing market registration as well as the discontinuation of traditional chemotherapeutics, incomplete insurance coverage for childhood cancers, bottlenecks in medicine procurement leading to (intermittent) stock-outs of essential medicines, low awareness on childhood cancers among primary healthcare staff as well as the general public and patients’ inability to access care facilities.

To complement the views and opinions of stakeholders, an understanding of the user perspective – or in this case the caregiver perspective – is pivotal (Thiede et al., 2007; Frenz & Vega, 2010). In prior research conducted globally, caregivers reported considerable financial and emotional difficulties in the diagnostic stages due to long delays and erratic referral pathways (Faruqui et al., 2019). Physical and emotional difficulties were also experienced during treatment, due to the chemotherapy, hospitalisation, and the drastic change in their lives (Alghamdi et al., 2023), as well as financial hardships (Islam et al., 2021; Sneha et al., 2017; Rativa Velandia & Carreño Moreno, 2018). In studies conducted in Sub-Saharan Africa, the need for financial assistance, clear information on the disease, emotional, spiritual and psychological support as well as material support was identified (Njuguna et al., 2015; Masika et al., 2020; Israëls et al., 2008; Kasahun et al., 2020). A single, small-scale study exploring the informational needs of South African parents identified a similar need for information about the diagnosis and treatment (Maree et al., 2016).

In South Africa almost 85% of the population relies on the government-funded public health sector; where health services and medications are provided at a nominal fee based on one's income, with exceptions for specific groups such as children under six and the economically disadvantaged (Suleman & Gray, 2017). The non-governmental organization (NGO) Children's Hematology Oncology Clinics (CHOC) Childhood Cancer Foundation provides additional support to caregivers reliant on public sector healthcare through the provision of free housing during a child’s course of treatment, as well as meals, toiletries, transport to healthcare facilities and small travel allowances (Childhood Cancer Foundation South Africa, 2023). Global evidence detailing the consequences of childhood cancer on caregivers (Faruqui et al., 2019; Alghamdi et al., 2023; Islam et al., 2021; Sneha et al., 2017; Rativa Velandia & Carreño Moreno, 2018; Njuguna et al., 2015; Masika et al., 2020; Israëls et al., 2008; Kasahun et al., 2020; Maree et al., 2016) may not directly translate to the South African context, such as support mechanisms provided by NGOs to caregivers in the public sector. Those who seek medical services at private sector clinics and hospitals typically cover their expenses through medical aid schemes (commonly known as health insurance) or face out-of-pocket (OOP) payments (Suleman & Gray, 2017). This sector is typically excluded from research into caregiver experiences (Rativa Velandia & Carreño Moreno, 2018; Njuguna et al., 2015; Masika et al., 2020).

Therefore, we sought to examine the experiences of caregivers in the South African context; with the aim of confirming the barriers and facilitators as perceived by professionals (Joosse et al., n.d), whilst identifying other potential determinants influencing caregiver experiences that previous stakeholder interviews have not uncovered. This context-specific evidence can contribute to the development of targeted policy interventions to facilitate improved access to childhood cancer care and reduce inequities in South Africa.

## Methods

Three semi-structured focus group interviews were conducted with groups of 4–10 caregivers of children with cancer in the South African public health care system. The scope of the present study was expanded to include the private sector in line with prior research activities (Joosse et al., 2023; Joosse et al. n.d). A single small focus group was conducted with caregivers from this sector.

### Participants

Caregivers were defined as any adult that accompanied and provided care for the child while receiving cancer treatment. Caregivers of children at any phase were eligible (diagnosis and staging, undergoing treatment, or treatment completed). Caregivers could include parents, grandparents, aunts and uncles, older brothers or sisters, or other persons living with and providing for the child. No particular exclusion criteria were applied, allowing any willing participant to join provided that they met the aforementioned definition of caregiver and had completed the informed consent document.

Public sector participants were recruited from the CHOC accommodation facilities in Durban (KwaZulu-Natal) and Cape Town (Western Cape) through convenience sampling. Prior to the sessions, caregivers were informed about the study by CHOC staff. On the day of the interview, caregivers present at the facility were informed about the aims and procedures of the study by the researchers and then invited to participate. All those invited agreed to participate.

Private sector participants were recruited through another non-profit organisation, which raises funds and awareness for families affected by childhood cancer. A foundation representative assisted in contacting caregivers involved with the foundation, approaching caregivers and informing them of the study. Six caregivers who had expressed interest in participating were forwarded to the researcher (IRJ), who contacted the caregivers to provide details about the aims and procedures of the study and invite them to participate. Attempts to schedule a focus group session were unsuccessful with four of the six caregivers.

### Procedures

The public sector focus groups were held in a common room at the respective CHOC accommodation facility in October 2022, whilst the private sector session took place online in November 2022. At the start of the interview, all participants gave their written consent to participate and completed a short questionnaire consisting of basic demographic data (such as age, sex, relation to the child, etc.). CHOC staff members or other participants assisted those of whom could not read or write in English sufficiently enough to complete the informed consent form and demographic survey for themselves. To avoid interference, researchers refrained from providing any assistance beyond the necessary clarifying information. Each participant was assigned a number to ensure their anonymity and to facilitate transcription of audio-recordings. Participants were asked about (1) the cancer journey, (2) the impact of the diagnosis and treatment on their lives and their family, (3) support, (4) experiences accessing care services, (5) financial experiences and costs made, and (6) unmet needs during the cancer journey (see Appendix 1). The interview guide was informed by prior research (Njuguna et al., 2015; Masika et al., 2020; Israëls et al., 2008; Kasahun et al., 2020; Maree et al., 2016) and was tested during the first session with caregivers, which led to minor modifications of the guide. All public sector sessions were moderated by IRJ (and FS), female academic researchers experienced in conducting interviews and with no prior connection to the participants. Sessions were conducted in the presence of a CHOC social worker or other staff members who could translate when the participant responded in Zulu (session 1) or Afrikaans (session 2). The third session was conducted in English/Xhosa whereby one of the participants also acted as translator. The private sector interviews were conducted online in English by IRJ. All interviews lasted for approximately 40 min.

### Data analysis

Audiotapes were transcribed verbatim and, when necessary, translated to English by a local translator. Transcripts were coded by IRJ and verified by FS to ensure that no themes were missed. Subsequent thematic analysis of the public sector sessions took place through a mixed approach, utilising a deductive component based on themes previously identified in literature (Sneha et al., 2017; Rativa Velandia & Carreño Moreno, 2018; Njuguna et al., 2015) alongside an inductive analysis following a grounded theory approach whereby data was coded iteratively to capture emerging themes. Data saturation was reached by the third session. An exploratory comparison was made between the themes identified in the public sector sessions and those that emerged within the private sector session.

### Ethics statement

Institutional approval was obtained from the Science-Geosciences Ethics Review Board (SG ERB) of Utrecht University (Bèta S-22784) and the Biomedical Research Ethics Committee (BREC) of the University of KwaZulu-Natal (BREC/00004635/2022).

## Results

A total of 20 caregivers from the public sector participated in the focus groups, of whom 15 participated actively in discussions via verbal contribution. One caregiver withdrew from their session due to overwhelming emotions. The caregivers that did not participate verbally during their session indicated their agreement by nodding and other non-verbal cues, but chose not to contribute verbally despite encouragement from the moderator to do so. The majority of caregivers identified themselves as either black (70%) or coloured (25%), with 65% being the mother of the child (see Table 1). There were two participants – both mothers – for the private sector session.

**Table 1.** General characteristics of participants.

	n	%	n	%
	Public sector participants		Private sector participants	
Number of participants	20	100	2	100
**Age group**				
15–24 years	3	15	0	0
25–34 years	9	45	0	0
35–44 years	4	20	0	0
45–54 years	2	10	2	100
> 55 years	2	10	0	0
**Gender**				
Male	1	5	0	0
Female	19	95	2	100
**Race**				
Black	14	70	0	0
Coloured	5	25	0	0
White	0	0	2	100
Unknown	1	5	0	0
**Relation to child**				
Parent	13	65	2	100
Grandparent	2	10	0	0
Other	5	25	0	0
**Age of child**				
0–2 years	4	20	0	0
3–5 years	4	20	0	0
6–8 years	6	30	0	0
9–12 years	4	20	0	0
12–15 years	2	10	2	100
**Highest level of education completed**				
Primary school	9	45	0	0
Secondary school	10	50	0	0
Diploma	0	0	1	50
University	1	5	1	50

## Public sector findings

**Figure 1.**
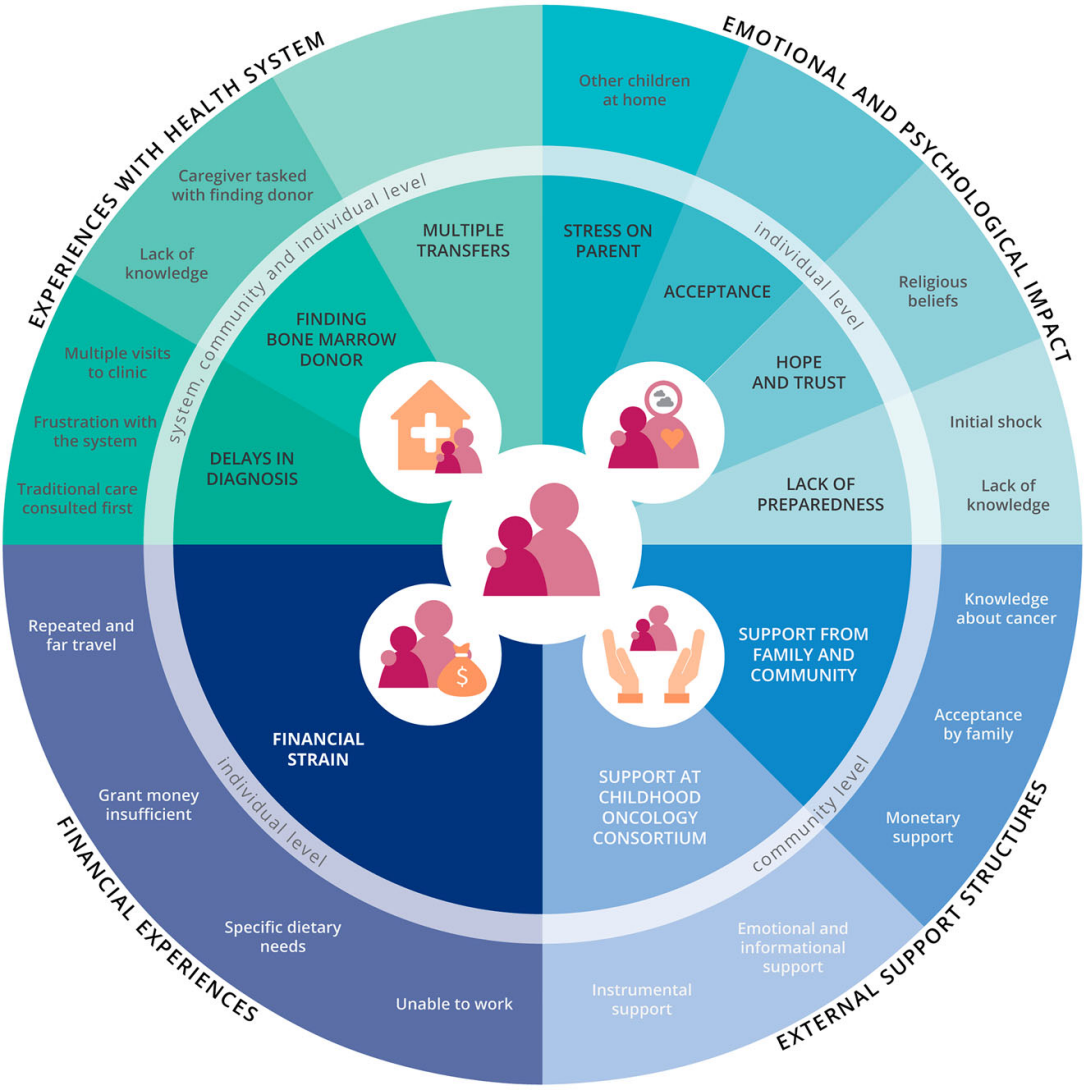
Caregiver experiences in accessing pediatric cancer treatment in the public sector. Each quadrant displays one of four main topics, including associated themes and subthemes, and at which level they occur.

Four major topics emerged from the data: (1) experiences with the health system, (2) emotional and psychological impact, (3) financial experiences and (4) external support structures. The issues and experiences associated with each of the main topics occurred at different societal levels, with ‘emotional and psychological impact’ and ‘financial experiences’ mainly occurring at the caregiver’s individual level, ‘external support structures’ exclusively taking place at a community level, and ‘experiences with the health system’ appearing at all levels (individual, community and (health) system’s level). An overview of all extracted themes and subthemes is provided in Figure 1.

### Experiences with the health system

Many caregivers shared experiences of testing and diagnoses being severely delayed, despite having made several visits to a primary healthcare clinic over multiple months before the child was finally referred for further testing to a hospital. Caregivers expressed their consequent frustration with the healthcare system and the primary point of care.

I started noticing that she was not okay in January this year I think. She started getting sick, fever, nausea. [I] took her to the clinic for like two months, until I went to the GP – a doctor – who told me to go immediately to the hospital. *P7, mother*

The sister then goes to another sister and says: ‘the child’s iron is low, why didn’t she come to the hospital?’ Then I said: ‘I came here for a long time, many years that I have been coming, the child has something wrong, and no one listened to me’. *P12, aunt*

Another participant shared that the diagnosis was initially delayed due to them seeking help from a traditional healer first:

It started with the child having pain in her eyes. We thought that it is our tradition, that maybe there is something we didn’t do, we did what we thought we should do. *P17, unspecified caregiver*

Caregivers described that the diagnosis and treatment required repeated referrals and transfers to different hospitals, often far away or even in a different province. The repeated travel, usually far away, imposed considerable financial strain on the family (see ‘financial strain’). The caregiver was compelled to be away from home for extended periods of time so as not to leave their child alone during treatment.

They admitted her for 2 months at Mandela, she was discharged on the 6th of September, then we went for check up on the 13th of September, then they said the cancer has spread inside the eye. And then we were transferred to Frere [Eastern Cape]. When we got to Frere, they checked her, they said her cancer is close to the brain, they said they don’t have treatment for her, she will be attended by doctors from here [Cape Town, Western Cape]. *P17, other caregiver*

Several caregivers shared their experiences with finding a bone marrow donor and undergoing subsequent transplants, indicating an (initial) lack of knowledge and understanding regarding donorship, coupled with the expectation to locate a suitable donor among family members. This imposed a further burden on the caregiver.

Her doctor told me [that] she must get a donor, [but] I know nothing about donors, because this is the first time to get cancer, to get a sick child like her. Her doctor told me I must look for a donor […]. *P19, mother*

### Emotional and psychological impact

Caregivers described feeling great shock and pain at the child’s diagnosis, compounded by a lack of knowledge on what the diagnosis entails and fearing for their child’s life. During their accounts of their experiences, several caregivers displayed strong emotions and expressed grief. One parent with intense emotions withdrew from the session at this stage.

When they told me at the hospital that the child has cancer, I cried. I cried because I didn’t know what cancer is like, because I would hear that cancer kills. *P8, unspecified caregiver*

[…] In the beginning it was too much for me to think of how I feel, it was hard, I didn’t know what it meant. *P13, mother*

Following the initial shock – having received more information on the disease and its prognosis – most caregivers expressed they had accepted the diagnosis.

I’ve accepted that my grandchild has cancer. But we were surprised because it’s for the first time we have someone who has cancer at home. *P1, grandmother*

In most caregivers, particularly in those whose children were further along in the treatment phase, acceptance was accompanied by hope and gratefulness. Many referred to their religious beliefs as an important source of hope.

Thank you that my child can see, thank you that my child has hope. Thank you for everything. You must just have that gratefulness. I don’t have to worry, there are angels, the angels will look after my children there. *P16, mother*

I don’t know how I will handle the aplastic anemia, but I do know one thing: God is with us. *P12, aunt*

Another great source of hope were the stories of other children at the ward and at the accommodation facility. One parent described that due to hearing other caregivers’ experiences she realised her own child’s situation was not as somber:

If you get some information about some other children, it was very hard for them to grow and be better, than mine was not that bad. So that gives you kind of hope that maybe she's gonna survive this. I have to be strong and just try and continue. *P7, mother*

Despite having accepted the diagnosis and having faith in a good outcome, nearly every caregiver expressed feeling burdened by the journey. Their stress was not only related to the child diagnosis and their suffering, but also due to having other duties besides taking care of the sick child. Having other children at home that they could not take care of was a significant stressor.

So, the journey is so traumatizing. […] Today she's feeling well, sometimes she's not feeling well. It's been ups and downs. […] As for myself, I think I need someone to talk to. Like a professional someone, to help me balancing things. I'm struggling to juggle between taking care of the kids and taking care of my schoolwork. So I even … I don't know how to cope. I don't know what to do anymore. I don't know where to start. So I'm struggling. *P3, mother*

### Financial experiences

Besides emotional stress, many caregivers experienced having difficulties coping financially as well. Despite the Childhood Cancer Foundation providing some monetary support to cover travel expenses to those living far away, the repeated travel to treatment facilities imposed great financial hardship on caregivers.

There are days that I struggle to get to the hospital. There were days that I did not have taxi fare, I made my way, I walked, the taxi dropped me, I had to walk very far to get to the hospital. […] Now in this month, […] this is my third week at the hospital. The blood [count] stays low. So for me, every week […] I must go see where I can get money […]. And next week I must be there again. *P13, mother*

Multiple caregivers reported that the Child Support Grant, which for some was the only source of income, remained insufficient to cover the additional expenses associated with cancer treatment. South African Social Security Agency (SASSA) Child Support Grant is a government grant intended to support lower-income households in South Africa in taking care of a child. At the time of interview, the grant amount was 450 ZAR (South African Rand; ± 25 USD) per child per month.

We come from far and the child support grant doesn’t help with anything, it gets used up on transport. The children can’t even have clothes because the money is used on transport to the hospital. *P4, mother*

Besides travel expenses amounting to significant additional costs, increased appetite and the need for special foods impose further financial pressure on the household.

If there’s food parcels, we would also like to receive them because we can’t afford. Since our children are sick, they need special diets to keep them healthy. So, we can’t afford that with the child support grant. *P8, unspecified caregiver*

Because what must your child eat? [What] your child is on, it makes them eat a lot. I did experience before the medications, she wasn't so fat. She's gaining weight now, like a lot. And because she's eating a lot, my cupboards, it's empty. […] Last night, it's like ‘mummy I'm hungry’. I couldn't offer her anything because I mean, there's nothing. And I did leave my job to look after her. And now who is there to provide? *P13, mother*

Lastly, economic hardship was exacerbated for some caregivers, who were forced to leave their jobs to accompany their child during treatment, resulting in a significant reduction in income. One parent reported being granted unpaid leave during the treatment period.

At work, they said […] family responsibility is only 5 days, no more than 5 days. They said they are going to give me unpaid leave yesterday. And then that is not easy for me because I used to have money. *P20, mother*

### External support structures

Social support from families and communities encountered in this study can broadly be categorised according to three types of assistance, (1) emotional, (2) instrumental (or tangible) and (3) informational support (Glanz et al., 2008). Caregivers had widely varying experiences in terms of receiving these support types from family and the community. While some received extensive support, others reported receiving no support at all.

My whole community supports me, my work supports me, my boss … […] They pray for my child, the church, every Wednesday evening they pray for her. I just have such a lot of support. *P16, mother*

Yes, there is support, from my parents, and my aunts, there aren’t other people who can support me besides them. *P17, unspecified caregiver*

Maybe for me, it is like people don’t like to help, they don’t like to be there, so I had to get used to that. *P13, mother*

Instrumental support from family and the community in some cases entailed monetary support:

For me, yes I did get support, my cousin’s sister did send for me something, money and also last year, they did give me some money and then this year also they did give me some money. *P20, mother*

From church, I do get something. *P18, grandmother*

That lack of support that some caregivers perceived was attributed to a lack of knowledge about cancer in the family and community. This perceptions seemed to stem from the misconception that the absence of severe symptoms implies the disease is non-serious.

I’m not getting support from family. They were surprised that the child has cancer. No one believes that the child has cancer because the child can walk, is not bed ridden. The child can walk. When I sleep in the hospital no one sees that this is serious. *P6, aunt*

Besides support from family and the community, there is considerable assistance provided through the Childhood Cancer Foundation. When discussing the foundation, the room's ambiance lifted noticeably, prompting some caregivers to laugh and smile. According to caregivers, emotional and informational support was not only provided through the foundation itself and its social worker in the ward, but also through other caregivers at the accommodation. Lastly, informational support was also provided by healthcare professionals.

But seeing that I'm not the only one going through this, sitting with these women here, honestly, it really does give me hope and keeps me strong because now I know I’m not alone. There are many of us. *P9, mother*

In addition to emotional and informational support, the Childhood Cancer Foundation provides critical instrumental support to caregivers.

Your CHOC house help us a lot for those who are far, you see, because they gave us toiletries and everything, food, a place to sleep, playing room for the kids. […] it is nice because they have transport for us, they have food for us. Sometimes here at hospital we don’t have food when we are there, we don't have food, at least here there is enough food for us. *P20, mother*

## Private sector findings

Experiences from caregivers in the private sector showed significant differences compared to caregivers seeking treatment in the public sector. This section describes the experiences of two private sector caregivers for each of the four main themes identified in the public sector.

### Experiences with the health system

Caregivers expressed great appreciation at the care received at their respective treatment centers. In contrast to public sector experiences, caregivers attested that symptoms were recognised immediately, resulting in swift intervention.

[Name of child] moaned about leg pain last November, we thought it was growing pains. But in January, it seemed to get worse. So we booked [an appointment] on the Friday I found a doctor, we had an appointment on the Monday, the Monday night the specialist phoned and the Tuesday we were admitted to hospital. *P22, mother*

Different to the public sector, caregivers described that they had the opportunity to utilise facilities and amenities provided by the hospital, including a bed or sleeper couch and food and drinks.

And the hospital was very, very accommodating. They had a parents menu as well. So I got three meals a day that was included as well. […] And then they also had mini fridges, where you could store some of your own things as well. So in terms of catering, it's made it a lot easier, especially for the kids because they can get quite picky. *P22, mother*

### Emotional and psychological impact

Psychological and emotional experiences were fairly similar to those in the public sector, with caregivers expressing distress at the diagnosis and the impact in their daily lives and that of their families. Hope, trust and acceptance were not prominently discussed, as both children had nearly completed their treatment and achieved positive outcomes.

### Financial experiences

Both families were members of a medical aid scheme and had invested in fairly comprehensive coverage prior to the diagnosis. As a result, insurance covered the majority of the medical expenses, except for blood transfusions. Neither caregiver reported substantial financial hardship due to the disease, in sharp contrast to the public sector caregivers.

So what my son's oncologist did is she worked out what the cost of the program would be more or less. So in the beginning, when [name of child] was first diagnosed, she motivated with [medical insurance scheme] and they made available 200,000 Rand for last year, and that pulled us through. *P21, mother*

### External support structures

Caregivers reported receiving considerable emotional support from their families and broader community, particularly from the schools. Neither caregiver required instrumental support from a non-profit organisation.

It was very uplifting to me to see the support that I got from the community. *P22, mother*

## Discussion

This study provides evidence of the emotional and financial challenges faced by caregivers in South Africa’s public healthcare setting when dealing with childhood cancer. The primary reasons for these hardships were the extensive travel expenses and associated prolonged periods of absence from home. While caregivers generally conveyed satisfaction with the care and support received at treatment facilities, they also expressed frustration towards the primary point of care in the public healthcare sector whereby delays in both testing and diagnosis were often remarked upon. The experiences of caregivers seeking care in the private sector confirmed the persistent inequities in the health system, with greater overall satisfaction with health services, financial protection from medical expenses and the resources to cover indirect costs such as travel expenses and dietary needs.

Our findings strongly align with prior research conducted among caregivers in the public sector in other African countries, reaffirming the previously reported lack of knowledge on childhood cancer, impact of repeated travel, financial difficulties, emotional and psychological burdens, along with the need for instrumental support (Njuguna et al., 2015; Masika et al., 2020; Israëls et al., 2008; Kasahun et al., 2020). However, in contrast to other recent studies, the support already provided by the Childhood Cancer Foundation in South Africa emerged as a critical contextual factor in the interpretation of caregivers’ financial experiences. Because despite offering some financial assistance and instrumental support (via the provision of food and accommodation) to caregivers, significant financial strain was still experienced. Furthermore, the foundation also extends emotional and psychological assistance through a dedicated social worker in the ward and their staff at the accommodation facility. While we emphasise the importance of continued emotional and psychological support from the foundation – as well as support from the caregiver’s own community – we identified no apparent need for additional support in this aspect. Similarly, the need for additional informational support was not directly raised (Maree et al., 2016). Healthcare professionals and the foundation together seem to be adept at foreseeing caregivers’ needs in this area. The significance of other parents within the ward was echoed in this regard (Njuguna et al., 2015). Notably, our study did not find evidence of unavailable medicines or supplies, nor a need for improved care at hospital level (Masika et al., 2020).

During focus group sessions with caregivers, no new major barriers to access emerged in comparison to interviews conducted with a range of stakeholders in South Africa’s pharmaceutical value chain; however, we were able to confirm firsthand the barriers that had previously been identified (Joosse et al., n.d). This indicates that healthcare providers and civil society in particular have a good understanding of user barriers. Nonetheless, this study sheds light on the emotional and psychological impact of childhood cancer on caregivers and provides a more nuanced insight into the extent of financial strain experienced by caregivers. The financial strain in this context is attributed to increased costs for travel and catering to the specific dietary requirements of the children; alongside this, there may be a (partial) loss of income, all the while balancing their responsibilities of supporting their families at home. Children exhibiting (hospital) food aversions or preferences was reiterated by caregivers in the private sector, underscoring the need for accustomed foods as an area of attention. The potentially far-reaching financial consequences of incomplete insurance coverage for those in the private sector (Joosse et al., n.d) could not be confirmed in this study, as both participants had taken out sufficient coverage.

To address the concerns and needs of caregivers of children with cancer in South Africa’s public sector, and reduce the inequities between both sectors, it is vital that families are financially protected from economic hardship through more adequate travel allowances and nutritional support. Healthcare professionals have previously indicated the need for a renewal of a temporary care grant system (Joosse et al., n.d), which can compensate families for increased costs and loss of income whilst undergoing cancer treatment. At present, only children with a permanent disability due to cancer, those who have undergone limb amputation or inoculation, are eligible for a care dependency grant. Additionally, the provision of cancer care closer to home and limiting transfers as much as possible could reduce the financial burden on families significantly, but potentially also lessen the impact of cancer treatment on their daily lives and increase access to care (Joosse et al., n.d). The need for expansions of services in regions with insufficient care provision was also recognised in the NCSF (National Department of Health, 2017); as was the need for improved training of primary care personnel on recognising childhood cancers. Finally, increased awareness on childhood cancers in the community may limit some of the emotional distress on caregivers and increase support.

Our findings recount the experiences of caregivers of children undergoing cancer staging or treatment. Our sample did not include caregivers of children who had completed their treatment. Despite this, the barriers we identified bears some significance for these survivors. Specifically, existing literature suggests that psychological distress in caregivers of childhood cancer survivors may persist long-term (Ljungman et al., 2014). Additionally, families of childhood cancer survivors reportedly often struggle with continued financial challenges due to ongoing follow-up care and poorer health (Nipp et al., 2017; Nathan et al., 2018). The needs and barriers experienced by childhood cancer survivors in South Africa and their families warrant further study.

An important strength of this study is that it allowed us to triangulate the experiences of caregivers with a broader health system analysis, confirming the user barriers as perceived by other stakeholders involved. Additionally, although the number of private sector participants was limited, this is to our knowledge the first study to include private sector caregivers, affirming and emphasising the differences between the public and private sectors. However, this small sample is likely not representative of all families seeking care in South Africa’s private sector, some of whom may not have adequate insurance coverage and could face catastrophic expenditures. We highlight this as an important area for further study.

This study is also subject to several limitations. Firstly, the recruitment of participants from the foundation's accommodation facility has led to selective recruitment, potentially missing caregivers with fewer needs for external support as well as families with no access to treatment or those defaulting. As a result, specific reasons for treatment abandonment are not fully captured in this study. However, the findings further contribute to building a foundational understanding of the factors involved (Israëls et al., 2008). Additionally, participating accommodation facilities were located in Cape Town and Durban and were linked to large tertiary and quaternary treatment centers. Experiences in other regions and smaller tertiary centers may be different, especially in regard to the availability of medicines and supplies. Finally, some participants had a greater contribution to the discussions while others were silent, with limited interactions between participants. This implies that some participants were uncomfortable sharing in this setting or on this (emotional) topic. This may have been particularly relevant for the male participant in an otherwise female-dominated session. This could have led to some experiences not being reported to the same extent as others. To mitigate potential participant barriers, the focus group interview setting was deliberately chosen to foster a sense of comfort among like-minded individuals, the sessions were conducted at the accommodation facility that was familiar to caregivers, and participants could respond to questions in their native language.

## Conclusion

This exploratory study provides evidence of user barriers in childhood cancer treatment, confirming and complementing findings from previous research. The experiences of caregivers in South Africa highlight that improvements are needed in the recognition of early signs of cancer at public sector primary care level. Despite indispensable emotional, informational and instrumental support provided by not-for-profit organisations, there is an increased need for financial support from the government through temporary social grants, travel allowances and nutritional support.

## Data Availability

The data that support the findings of this study are not publicly available. Anonymized data are available from the authors upon reasonable request.

## Appendix 1

### Semi-structured guide for focus group sessions

#### General questions

Can you tell us a little about your family and your experience with the disease and care of your child?

Can you tell us what the disease has meant for you and your family?

#### Support

Is anyone supporting you or your family?

#### Accessing care and medicines

Do you know of any experience or story where a caregiver had issues accessing cancer services or medicines for their child? Please tell us more about that.

#### Financial aspects

We would now like to ask you a few more questions about the costs of cancer care for children.

What can you tell us about the costs caregivers have to make for their child’s cancer care?

- Probes: consultation costs, investigation/imaging costs, medicines cost, transportation/travel costs, food and other related costs.
- Is there anything that the hospital has provided for you and your child? If yes, what? (Meals, fruit, travel costs, place to stay, other)
- Do you have medical insurance? If so, what did it (not) cover? (*private sector only*)

#### Closing

What do you need or what did you need at any point in your journey to make it better to make it easier?

Is there anything that you would like to say that I haven’t already asked?
